# Inclusive inquiry: a compassionate journey in trauma-informed qualitative research with GBV survivors from displaced communities

**DOI:** 10.3389/fpsyg.2024.1399115

**Published:** 2024-07-25

**Authors:** Jasmin Lilian Diab, Dana Al-Azzeh

**Affiliations:** ^1^School of Arts and Sciences, Institute for Migration Studies, Lebanese American University, Beirut, Lebanon; ^2^Department of Social and Education Sciences, School of Arts and Sciences, Lebanese American University, Beirut, Lebanon

**Keywords:** trauma-informed, gender-based violence, displacement, qualitative approaches, research

## Abstract

The integration of trauma-informed and ethical frameworks in qualitative research concerning survivors of gender-based violence (GBV) within displaced communities is critical. These individuals often bear the weight of traumatic experiences compounded by displacement and associated hardships. Adopting a trauma-informed approach establishes a safe environment, prioritizing survivors’ well-being and respecting their agency and narratives, thereby fostering trust and reducing re-traumatization risks. Ethical considerations ensure the dignity, rights, and cultural sensitivities of participants are upheld, contributing to rigorous and humane research. This integration amplifies survivors’ voices and experiences, enhancing understanding and empathy. Trauma-informed approaches acknowledge the likelihood of trauma in individuals’ lives and prioritize safety without aiming to treat symptoms. Proficient interviewing skills aim to improve comfort, safety, and recall without avoiding challenging questions. Integration of trauma-informed principles across all interview phases is crucial, particularly for individuals experiencing various traumas simultaneously, such as displacement, violence, and ongoing conflict. Drawing from the authors’ experiences and existing literature, this paper advocates for a compassionate and empowering shift in qualitative research methodologies to better engage with survivors of trauma and GBV within displaced communities.

## 1 Introduction

Anchoring qualitative research within a trauma-informed and ethical framework is of paramount importance when engaging with survivors of gender-based violence (GBV) from displaced communities. These individuals often carry the heavy burden of traumatic experiences and are navigating the complex interplay of emotional and psychological scars as a result of displacement and the associated hardships they have endured. By adopting a trauma-informed approach, researchers can create a safe and supportive environment for survivors, prioritizing their emotional and physical well-being while also respecting their agency and narratives. This not only fosters trust and empowers survivors to share their stories but also reduces the risk of re-traumatization during the research process (Ghidei et al., 2022). An ethical foundation ensures that the dignity, rights, and cultural sensitivities of the participants are respected and that their voices are heard authentically, contributing to the creation of research that is both rigorous and humane.

The integration of a trauma-informed, ethical approach in qualitative research is essential in amplifying the voices and experiences of survivors from displaced communities and promoting a more empathetic and comprehensive understanding of their realities. A trauma-informed approach is “[…] an approach in the human service field which assumes that an individual is more likely than not to have a history of trauma and acknowledges the impact trauma may have on someone’s life” (Institute on Trauma and Trauma-Informed Care, 2015). Trauma-informed approaches do not aim to treat symptoms associated with trauma, but rather prioritize the psychological and physical safety of individuals (Azzeh, 2023). They employ evidence-based approaches to prevent the re-traumatization of survivors of violence, specifically those who have experienced GBV (Isobel, 2021). Importantly, practicing trauma-informed interviewing does not entail avoiding difficult questions; instead, it involves posing challenging questions in a sensitive manner to safeguard against re-traumatizing the survivor. Proficient interviewing skills with GBV survivors pursue two essential goals: elevating the level of comfort and safety for survivors and improving the survivor’s recall of the traumatic incident (Isobel, 2021).

It is crucial to emphasize that trauma-informed principles should be integrated across all phases of the interview, spanning from preparation to closure. From this standpoint, the article seeks to explore, in an intersectional manner, the significance of this approach when individuals undergoing interviews are concurrently dealing with diverse forms of trauma, including displacement, surviving violence, and enduring the long-term consequences of an ongoing conflict. This paper builds on an article series on ‘Trauma, GBV and Refugeehood’ published by the Institute for Migration Studies at the Lebanese American University, (Darouiche, 2023) as well as the co-authors’ personal experiences undertaking qualitative research at this intersection (Diab et al., 2023). This paper advocates for a paradigm shift in qualitative research methodologies to ensure a more compassionate and empowering engagement with displaced individuals who have experienced trauma and GBV. As such, the paper journeys through discussing the prevalence of trauma and GBV among displaced populations, the importance of trauma-informed approaches in understanding and framing the survivors’ experiences, as well as the barriers to carrying out this type of research, positionality of the researcher, ethical imperatives, as well as the importance of minimizing harm. It moves on to unpacking the importance of survivor-centered narratives and participatory action research, to conclude with recommendations for researchers endeavoring to explore this space and intersection.

## 2 The prevalence of trauma and GBV among displaced populations

Displaced populations, including refugees and internally displaced persons (IDPs), face a heightened risk of experiencing trauma and GBV due to the disruptive and often perilous nature of forced migration (Wirtz et al., 2014). The prevalence of trauma and GBV within these populations is a complex and multifaceted issue shaped by the intersection of conflict, displacement, and cultural dynamics. Trauma among displaced groups is understood across two important fronts: conflict-induced trauma (Dumke et al., 2021) and forced displacement trauma (Dowd, 2020). For many displaced individuals, conflict-induced trauma includes exposure to violence, loss of loved ones, and the experience of displacement itself (Dowd, 2020). Moreover, the ongoing threat of harm, displacement-related stressors, and the loss of a familiar environment exacerbate the psychological toll (Dowd, 2020). When it comes to forced displacement trauma, the act of being forcibly uprooted from one’s home and community, the uncertainty of the future, loss of identity, and challenges in adapting to new environments further contribute to the mental health burden among displaced populations (Dowd, 2020).

### 2.1 GBV in exacerbating pre-existing vulnerabilities and perpetuating new ones

In the challenging landscape of displacement, the specter of GBV looms larger, exacerbating pre-existing inequalities and gender dynamics (Raftery et al., 2023). Displaced women, girls, and other vulnerable groups find themselves more susceptible to a range of violence, from sexual violence to intimate partner violence and exploitation (Raftery et al., 2023). The settings of displacement camps and temporary shelters, intended as havens, paradoxically become breeding grounds for GBV (Raftery et al., 2023). Overcrowded conditions, inadequate lighting, and limited security measures heighten vulnerability, fostering an environment conducive to the flourishing of violence. GBV often occurs in communities where fetching essential resources such as water necessitates long walks, primarily affecting women and girls (Global Protection Cluster and Inter-Agency Standing Committee, 2015). The arduous journey to access these resources exposes them to various risks, including harassment, assault, and exploitation (Global Protection Cluster and Inter-Agency Standing Committee, 2015). Furthermore, the placement of communal bathrooms at distant locations from the tents exacerbates the vulnerability of women and girls, particularly during the night, when traversing long distances becomes perilous (Oxfam and WEDC and Loughborough University, 2017). Simultaneously, the breakdown of traditional social structures contributes to an alarming increase in both domestic and communal violence. Displacement not only displaces individuals physically but also exposes them to elevated risks of human trafficking and exploitation (Cockbain and Sidebottom, 2022). The vulnerability of displaced populations, coupled with the breakdown of protective mechanisms, creates a perfect storm for various forms of human rights abuses. While GBV is indeed a pervasive issue in urban settings, exacerbated by various factors such as crowded living conditions, inadequate infrastructure, economic disparities, and social inequalities, (Cameron and Tedds, 2021) in tented settlements and refugee camps, the situation is often exacerbated and more severe due to several challenges including inadequate shelter, poor infrastructure, inadequate access to services and heightened dependency and competition over humanitarian aid (Dahie et al., 2022).

Examining the intersectionality of trauma and vulnerability unveils distinct experiences among different demographic groups. Women, children, and members of the LGBTIQ+ community navigate their own traumas (Alessi et al., 2018). Children grapple with neurological developmental challenges gender inequities, xenophobia and racial discrimination, (Hazer and Gredebäck, 2023) while women and non-binary individuals confront gender-specific violence, discrimination, and exploitation (Hazer and Gredebäck, 2023). Cultural dynamics further complicate this issue. Cultural factors and norms become crucial determinants in either exacerbating or mitigating trauma and GBV within displaced communities (Ford et al., 2015). The stigmatization of survivors, cultural expectations surrounding gender roles, and adherence to traditional practices emerge as influential factors that either perpetuate or challenge various forms of violence (Lanchimba et al., 2023).

One factor in mitigating the impact of trauma on individuals, particularly in settings like tented settlements and refugee camps where resources and security are limited, is communal support (Sabri and Granger, 2018). When communities come together to provide support, empathy, and solidarity to survivors of traumatic incidents, it can significantly aid in their recovery process. Judith Herman, in her seminal work ‘Trauma and Recovery,’ extensively discusses the importance of social connections and communal support in healing from trauma (Kristen et al., 2016). Herman’s work emphasizes the importance of creating supportive environments where survivors feel heard, valued, and empowered to heal (Kristen et al., 2016). Communal support plays a pivotal role in aiding survivors of trauma by providing validation and belief in their experiences, fostering a sense of safety and protection, and normalizing their responses to traumatic events (Kristen et al., 2016). When individuals are heard and believed by their community, it can alleviate feelings of self-blame, shame, and isolation commonly associated with trauma. Moreover, the presence of a supportive community offers survivors a secure environment to process their emotions, and ensures practical assistance from communities such as facilitating access to medical care or counseling services (Idriss-Wheeler et al., 2022). Cultural and spiritual practices within communal support further offer comfort and connection, aiding in healing and resilience-building (Pertek et al., 2023).

In the absence of communal support, survivors may experience increased isolation, heightened vulnerability to further trauma, and delayed recovery from post-traumatic stress disorder (PTSD) (Calhoun et al., 2022). The absence of protective networks has been found to leave individuals vulnerable to exploitation and re-victimization, exacerbating their trauma (Calhoun et al., 2022). The urgent nature of trauma and GBV among displaced populations necessitates comprehensive, trauma-informed interventions. These interventions must intricately address the unique needs of individuals who have experienced displacement. Recognizing the intersectionality of trauma and GBV, along with a nuanced understanding of the cultural contexts in which they unfold, is paramount in designing effective and sensitive support systems for displaced communities.

### 2.2 Trauma-informed, ethical, and qualitative approaches in understanding and framing the survivor experience

Understanding the nuanced and often silenced experiences of GBV survivors is essential for informing effective interventions and support systems. The significance of qualitative research in shedding light on the narratives of GBV survivors emphasizes the necessity of a trauma-informed, ethical approach to ensure the well-being of those who choose to share their stories. GBV encompasses a range of physical, sexual, emotional, and economic abuses that are deeply intertwined with power imbalances, societal norms, and cultural expectations (Perrin et al., 2019). Each survivor’s journey is a unique tapestry of trauma, resilience, and survival, making it imperative to move beyond statistical data and delve into the qualitative dimensions of their experiences. While quantitative data provides valuable insights into the prevalence and patterns of GBV, it often falls short in capturing the rich complexity of survivors’ lived experiences (Testa et al., 2011). Qualitative research methods offer a deeper understanding by exploring the context, emotions, and individual perceptions that quantitative data might overlook (Haradhan, 2018). A qualitative trauma-informed approach is paramount in researching GBV experiences. Survivors often carry the weight of their trauma, and interviews must be conducted with utmost sensitivity and awareness of potential triggers. Trauma-informed qualitative research recognizes the impact of trauma on individuals and structures research methodologies accordingly, prioritizing safety and empowerment (Isobel, 2021).

Ethical guidelines are crucial in safeguarding the well-being and dignity of GBV survivors participating in research (Mootz et al., 2019). Informed consent processes must be comprehensive, ensuring participants understand the nature of the study, potential risks, and their right to withdraw at any time (Mootz et al., 2019). Confidentiality and privacy measures are equally vital in protecting survivors from unintended harm. Along these lines, survivor-centered methodologies shift the focus from traditional researcher-driven approaches to empowering survivors as active participants in the research process (Jumarali et al., 2021). This approach respects survivors’ agency, allowing them to shape the narrative and control the pace and depth of disclosure. Through this lens, the research has the power to amplify the voices of marginalized GBV survivors who often face intersectional challenges such as racism, economic disparities, and discrimination based on sexual orientation or gender identity. By embracing diverse perspectives, this approach contributes to a more inclusive understanding of GBV as well as the narratives that shape it (Jumarali et al., 2021).

In addition to its role in understanding individual experiences, qualitative research informs the development of policies and practices that address the root causes of GBV (Ford and Annelies, 2021). By uncovering systemic issues and cultural dynamics, research findings can guide the creation of more effective prevention and intervention strategies. Qualitative research stands as a crucial tool in the pursuit of comprehensive insights into the experiences of GBV survivors. By adopting trauma-informed and ethical approaches, researchers can honor survivors’ narratives, contribute to a deeper understanding of the complexities surrounding GBV, and pave the way for more compassionate and effective interventions in the fight against GBV.

## 3 Unpacking trauma and refugeehood

Trauma, within the context of refugeehood, is a multifaceted and profound force that leaves indelible marks on both individuals and the communities they form. The displacement, violence, and loss inherent in forced migration contribute to a complex web of psychological, emotional, and social challenges that demand careful consideration (Cuadrado et al., 2023). For refugees, trauma often begins with the experiences that led to their displacement. Exposure to conflict, persecution, and the loss of homes and loved ones create a profound sense of vulnerability (Silove et al., 2017). The journey itself, marked by perilous escapes and uncertain futures, amplifies the trauma. The psychological toll of these experiences can manifest as anxiety, depression, post-traumatic stress disorder (PTSD), and other mental health conditions, affecting individuals’ ability to rebuild their lives (Venkatachalam et al., 2023).

Trauma is not isolated to individuals; it permeates the fabric of communities. Forced migration disrupts social structures, fractures familial bonds, and erodes the communal networks that are crucial for coping and resilience (Bunn et al., 2023). The loss of cultural identity, traditions, and a sense of belonging further compounds the collective trauma experienced by refugee communities (Alexander et al., 2004). This interconnected trauma can create a shared narrative of suffering that shapes the community’s outlook and its members’ interactions (Alexander et al., 2004). For this reason, understanding trauma within the context of refugeehood requires cultural sensitivity (Im and Swan, 2021). Trauma may be expressed differently across diverse cultural backgrounds, influencing coping mechanisms and help-seeking behaviors (Schnyder et al., 2016). Cultural factors, including religious beliefs, traditional healing practices, and community support systems, play a pivotal role in shaping how individuals and communities navigate the aftermath of traumatic experiences (Maier et al., 2022). Knowledge of how this plays out inter-generationally is pivotal. Children in refugee communities are particularly vulnerable to the long-term effects of trauma (Fegert et al., 2018). Displacement disrupts their education, exposes them to violence, and hampers their social development (Fegert et al., 2018). The trauma experienced during formative years can have lasting consequences on mental health, educational attainment, and overall well-being. Furthermore, the transmission of trauma across generations is a recognized phenomenon, as the experiences of parents can shape the mental health and resilience of their children (Kizilhan et al., 2021).

While trauma poses significant challenges, resilience also emerges as a defining characteristic of many refugee communities (Gatt et al., 2020). Individuals and communities draw on their internal strengths, cultural resources, and support networks to navigate the complexities of trauma (Gatt et al., 2020). Recognizing and fostering resilience becomes crucial in crafting effective interventions and support systems that acknowledge the agency and strengths of refugees in rebuilding their lives. A nuanced understanding of trauma’s impact is essential for developing interventions that are culturally sensitive, trauma-informed, and capable of fostering resilience among those who have experienced the profound disruptions of forced migration.

## 4 Examining specific challenges and manifestations of GBV in displaced communities

GBV in displaced communities constitutes a complex web of challenges, its manifestations intertwined with the unique circumstances of forced migration, cultural dynamics, and the breakdown of traditional support structures (Doron, 2005). Understanding the nuanced challenges faced by individuals in these contexts is imperative for devising effective interventions and support systems. The upheaval of normal life, loss of protective community structures, and the crowded conditions in camps or temporary settlements create an environment where perpetrators may exploit power imbalances with greater impunity (McAlpine et al., 2020).

The stressors associated with forced migration, including uncertainty about the future and economic instability, can exacerbate tensions within households, contributing to an increase in violence within intimate relationships (McAlpine et al., 2020). The displacement of families due to conflict often leads to significant shifts in household dynamics, particularly regarding the role of women as breadwinners (Horn et al., 2014). In many cases, as men may face barriers to employment or may be absent due to conflict-related factors, women often assume the role of primary earners within refugee households (Horn et al., 2014). While this transition can empower women economically, it can also exacerbate existing power imbalances within the family structure. Traditional gender roles and expectations may clash with the new reality of women as breadwinners, leading to tensions and conflicts within the household (Mehra et al., 2023). This shift challenges traditional notions of masculinity and femininity, potentially undermining the patriarchal authority that previously governed family dynamics (Perrin et al., 2019). As a result, men may feel emasculated or threatened by the loss of their traditional role as providers, leading to feelings of resentment or inadequacy that can manifest in abusive behaviors towards women and children (Perrin et al., 2019).

The vulnerability of displaced populations makes them susceptible to exploitation and human trafficking. Women and children, in particular, may be targeted for forced labor, sexual exploitation, or other forms of trafficking (Bassiouni et al., 2010). The breakdown of familiar social structures and the economic desperation that often accompanies displacement create conditions ripe for exploitation. Certain groups within displaced communities, such as LGBTIQ+ individuals, may face heightened discrimination and marginalization (Diab et al., 2024). The intersectionality of gender with other identities further complicates the challenges faced by individuals, as they may be subjected to violence based not only on their gender but also on their sexual orientation, gender identity, or other social factors (Diab et al., 2024).

Displaced communities often have limited access to support services, including healthcare, counseling, and legal assistance (Diab et al., 2024). The stigmatization of survivors, coupled with the absence of culturally sensitive services, may deter individuals from seeking help. Barriers such as language differences and a lack of awareness about available resources further compound these challenges. The very act of displacement inflicts trauma, and this trauma can be a precursor to or exacerbate GBV. The uncertainty, loss, and upheaval experienced during forced migration can contribute to mental health issues, which, in turn, may make individuals more susceptible to different forms of GBV (Fouad et al., 2021). The challenges and manifestations of GBV in displaced communities remain deeply rooted in the complex dynamics of forced migration. Addressing these challenges requires a comprehensive and culturally sensitive approach that not only responds to immediate needs but also acknowledges the long-term impact of displacement on the dynamics of gender-based violence. Developing targeted interventions that account for the specific challenges within displaced communities is essential for fostering safety, empowerment, and resilience among survivors of GBV.

### 4.1 Societal attitudes and prejudices impacting survivors’ willingness to participate in research

Research on GBV and trauma heavily relies on the willingness of survivors to share their experiences. However, societal attitudes and prevailing prejudices can significantly influence survivors’ decisions to engage with research initiatives. Understanding these dynamics is pivotal for researchers seeking to conduct ethical and trauma-informed studies. Societal stigmatization surrounding GBV survivors can contribute to a pervasive culture of shame and blame. Survivors may fear judgment, ostracization, or the reinforcement of negative stereotypes if they disclose their experiences. The fear of being labeled as a victim or being perceived as somehow responsible for the violence they endured can be a powerful deterrent to participation in research. To mitigate survivors’ fears and concerns regarding participation in research, researchers can prioritize building trust and creating safe spaces for disclosure (Ford et al., 2009). This can be achieved through transparent communication about the purpose and goals of the research, emphasizing confidentiality and anonymity protections, and providing clear information about how data will be used and stored securely (Ford et al., 2009). Researchers should also demonstrate sensitivity and empathy towards survivors’ experiences, ensuring that they feel respected and supported throughout the research process (Anderson et al., 2023). Additionally, involving survivors in the design and implementation of research studies can empower them and help to ensure that their perspectives and needs are adequately addressed (Thomas et al., 2022). By actively addressing survivors’ concerns and prioritizing their well-being, researchers can foster a supportive environment that encourages participation and contributes to a more comprehensive understanding of GBV.

Cultural norms and taboos surrounding discussions of intimate matters or GBV can impede survivors from openly sharing their experiences. Societal expectations regarding privacy and familial honor may discourage individuals from speaking out, fearing repercussions not only for themselves but for their families as well. The prospect of breaching these cultural norms may hinder survivors’ willingness to participate in research. In societies where patriarchal structures are deeply ingrained, survivors may fear retaliation from perpetrators or their social networks if they disclose incidents of GBV. The potential consequences, ranging from social isolation to physical harm, can be significant barriers to participation in research. The lack of protective mechanisms within communities may exacerbate survivors’ concerns about safety. To address these concerns, researchers can implement several strategies. Firstly, researchers should prioritize safety and confidentiality by ensuring that all data collection processes are conducted discreetly and securely (Kaiser, 2009). This may involve using anonymous surveys or conducting interviews in private settings where survivors feel safe to share their experiences without fear of repercussions. Additionally, researchers can collaborate with local organizations and community leaders to establish support networks and protective mechanisms for survivors, providing resources such as legal aid, counseling services, and safe shelters through referrals. By actively involving the community in research efforts and advocating for survivors’ rights, researchers can help to mitigate fears of retaliation and create a more supportive environment for disclosure and participation.

Survivors who belong to marginalized or minority groups may face intersectional challenges, with societal prejudices compounding their reluctance to participate. Individuals who experience discrimination based on factors such as race, ethnicity, sexual orientation, or socioeconomic status may anticipate additional layers of bias, making them wary of engaging with research initiatives. Historical and systemic factors can contribute to a general distrust in institutions, including research organizations. Survivors may be skeptical about how their stories will be utilized, whether their confidentiality will be maintained, or if their experiences will be exploited for academic or sensational purposes. A lack of trust in the research process may lead survivors to avoid participation altogether. To address these concerns, researchers must prioritize transparency and ethical conduct. This not only involves clearly communicating the purpose and objectives of the research, but more specifically how survivors’ narratives will be used, ensuring that their confidentiality is rigorously maintained through anonymization and data protection measures (Kaiser, 2009). Researchers should also emphasize the importance of survivor autonomy and consent, allowing individuals to decide how much information they are comfortable sharing and respecting their right to withdraw from the study at any time (Seagle et al., 2020). Building trust with survivors requires establishing rapport, demonstrating empathy, and actively listening to their concerns (Seagle et al., 2020). By fostering an environment of openness and accountability, researchers can mitigate skepticism and encourage survivors to participate in research efforts aimed at addressing GBV effectively (Seagle et al., 2020).

Media portrayals and sensationalist narratives surrounding GBV can shape public perceptions and influence survivors’ expectations of research endeavors. The fear of exploitation or the re-traumatization that may result from insensitive or sensationalized representations can discourage survivors from engaging with researchers (Anderson et al., 2023). The underrepresentation of diverse voices and experiences within research can perpetuate mistrust. If survivors do not see themselves reflected in the researchers, if the research team lacks cultural competence, or if studies fail to encompass a broad range of experiences, potential participants may question the relevance and authenticity of the research. Societal attitudes and prejudices play a pivotal role in shaping survivors’ willingness to participate in research on GBV. Researchers must recognize and address these barriers, fostering an environment of trust, cultural sensitivity, and inclusivity. A commitment to dismantling stigma, empowering survivors, and respecting diverse identities is crucial for establishing ethical and survivor-centered research practices.

### 4.2 Power imbalances between researchers and participants, and their potential impact on the research process

Research, particularly in sensitive areas such as GBV, operates within a dynamic framework of power relations. The inherent power imbalances between researchers and participants have profound implications for the research process, influencing everything from the initial engagement to the dissemination of findings. Acknowledging and mitigating these power dynamics is essential for ethical, respectful, and inclusive research practices. Power imbalances can impede the truly voluntary nature of informed consent. Participants may feel pressure to participate due to perceived authority or fear of repercussions. As discussed, researchers must be mindful of ensuring that participants feel genuinely empowered to make autonomous decisions about their involvement in the study. The framing of research questions and the overall research agenda are critical aspects of the research process. The inherent power of researchers to set these parameters can unintentionally lead to a skewed perspective that may not fully capture the priorities or concerns of the participants. Inclusive and collaborative approaches, similar to the participatory approach previously discussed, are necessary to mitigate this imbalance (Duea et al., 2022).

During data collection, the power dynamic between researchers and participants significantly influences both the framing of questions and the nature of interactions (Kaaristo, 2022). This influence is evident in various aspects of the data collection process. For instance, researchers may unintentionally pose leading questions that suggest a particular response or bias participants’ answers, thus shaping the narrative in line with the researchers’ assumptions. Moreover, the choice of language and terminology used in questions can reflect underlying power differentials, with certain phrasings reinforcing stereotypes or victim-blaming narratives (Mats and Steinvall, 2020). Researchers’ control over the conversation during interviews or focus groups further underscores this power dynamic, as they may steer discussions toward topics of interest or silence participants who raise uncomfortable points, limiting their autonomy and agency in shaping the conversation.

Furthermore, implicit biases of researchers can permeate the data collection process, influencing question framing and interpretation (Wilson et al., 2022). Researchers may inadvertently reflect their own biases and preconceptions in the questions they ask, (Holmes, 2020). potentially perpetuating harmful stereotypes or overlooking alternative perspectives (Galdas, 2017). Additionally, cultural sensitivity plays a crucial role in shaping the framing of questions, as researchers from different cultural backgrounds may unintentionally pose questions that are insensitive or offensive to participants, hindering effective communication and rapport building (Liamputtong, 2010). An awareness of this dynamic is crucial for maintaining the integrity of participant voices. Participants, often in vulnerable positions, may feel compelled to provide responses they believe researchers want to hear or that align with societal expectations. This can lead to skewed data and a lack of authenticity in the narratives shared. As outlined, researchers must create an environment where participants feel safe to express their genuine experiences.

Researchers hold the power to provide resources, opportunities, or access to services for participants (Santoro, 2023). This dynamic can create a sense of dependency or expectation among participants, influencing the nature of their interactions with the research team. Researchers must be transparent about the limitations of what they can offer and manage expectations accordingly (Santoro, 2023). The power dynamic continues into the dissemination phase. Researchers, as authors and disseminators of findings, possess the authority to shape narratives and control the representation of participants’ experiences. The power dynamics persist beyond the completion of the research project. Power imbalances between researchers and participants are inherent in the research process, and their impact can be far-reaching (Råheim et al., 2016). Vigilance, reflexivity, and a commitment to equitable and inclusive practices are essential for navigating these dynamics responsibly (Råheim et al., 2016). By fostering collaborative relationships, researchers can contribute to more ethical, respectful, and impactful research outcomes, particularly in areas as sensitive as the study of gender-based violence (Råheim et al., 2016).

## 5 The ethical imperative of trauma-informed research

The cornerstone of ethical engagement with participants when considering trauma-informed research, is the informed consent process. Recognizing the unique vulnerabilities and sensitivities of individuals who have experienced trauma, ensuring clear and comprehensive informed consent is paramount for upholding the principles of respect, autonomy, and safety throughout the research journey (Risan et al., 2020). Informed consent is more than a procedural formality; it is a mechanism for empowering survivors. Trauma often involves a sense of disempowerment and loss of control. A transparent and comprehensive informed consent process equips survivors with the knowledge and agency to actively engage in the research, fostering a sense of control over their participation (Risan et al., 2020).

Importantly, assuming that individuals with a history of trauma are incapable of understanding the research process or giving informed consent can perpetuate dynamics of disempowerment and re-traumatization, contradicting trauma-informed principles. Trauma-informed research practices emphasize the importance of respecting individuals’ autonomy, agency, and capacity for decision-making, regardless of their past experiences (Risan et al., 2020). By presuming that trauma survivors lack the ability to comprehend the research process or provide consent, researchers risk perpetuating harmful dynamics of paternalism and disempowerment, which can replicate the power imbalances that often characterize traumatic experiences (Herman, 2015). Such assumptions undermine the principles of empowerment and collaboration inherent in trauma-informed approaches, denying survivors the opportunity to actively participate in research processes and contribute their perspectives. Instead, trauma-informed research should prioritize creating safe and supportive environments that facilitate informed decision-making and respect survivors’ agency, recognizing their capacity for understanding and engagement in the research process (Goelitz, 2020).

Trauma survivors may grapple with heightened vulnerability and sensitivity to potential triggers. A well-constructed informed consent process provides clear and accessible information about the research, its objectives, and potential outcomes. This clarity aids survivors in making informed decisions, minimizing anxiety, and creating an environment conducive to open communication (Gillihan, 2023). Trauma survivors may have unique boundaries and thresholds for engaging in discussions about their experiences. A trauma- informed consent process acknowledges and respects these boundaries. It allows survivors to set parameters on the extent of their participation, ensuring that they remain in control of the information they choose to disclose (Gillihan, 2023). The informed consent process should be viewed as an ongoing dialogue rather than a one-time event. Regular and open communication between researchers and participants helps maintain a responsive and supportive environment throughout the research journey (International Association of Chiefs of Police [IACP], 2017). Trauma survivors may have specific triggers that evoke distressing memories or emotions. The informed consent process becomes a crucial tool for identifying and addressing potential triggers. By openly discussing potential sensitive topics and offering participants the choice to skip or modify certain questions, researchers contribute to a more compassionate and minimally distressing research experience (International Association of Chiefs of Police [IACP], 2017).

Trauma survivors often have heightened concerns about privacy and confidentiality. The informed consent process should explicitly outline the measures in place to safeguard participants’ information, assuring them of the confidentiality of their disclosures. This assurance fosters a climate of trust, encouraging survivors to share their experiences more openly. The language used in the informed consent documents and discussions should be trauma-informed. Clear, plain language should be employed, and potential jargon or clinical terms should be explained to ensure participants fully comprehend the information presented. This approach aligns with the trauma-informed principle of creating a safe and accessible space for survivors.

Beyond ethical considerations, the informed consent process provides legal protections for both researchers and participants. By clearly outlining the rights and responsibilities of all parties involved, the informed consent document serves as a foundational document that upholds the integrity and legitimacy of the research. A robust informed consent process is not only an ethical imperative in trauma-informed research but also a means to honor and prioritize the well-being of survivors (International Association of Chiefs of Police [IACP], 2017). By promoting transparency, respecting boundaries, and maintaining continual communication, researchers can create an environment that fosters trust, autonomy, and a more inclusive and empowering research experience for individuals who have experienced trauma (International Association of Chiefs of Police [IACP], 2017).

### 5.1 Addressing the need for robust measures to protect participants’ confidentiality and privacy

When delving into sensitive topics such as trauma or gender-based violence, safeguarding participants’ confidentiality and privacy is not just a procedural requirement but an ethical imperative. The commitment to robust measures in this regard is foundational to fostering trust, ensuring participant well-being, and upholding the integrity of the research process. Confidentiality forms the bedrock of trust between researchers and participants. Trauma survivors or individuals disclosing sensitive information must feel assured that their trust is not misplaced. A breach of confidentiality can have profound consequences, eroding trust not only in the research process but potentially in seeking support or sharing experiences in the future. Research on trauma or gender-based violence often involves engaging with vulnerable populations (Kaiser, 2009). These individuals may already be navigating complex circumstances, and any compromise of their confidentiality could expose them to further risks, ranging from social stigma to potential harm from perpetrators. Recognizing these risks underscores the gravity of maintaining robust confidentiality measures.

The informed consent process plays a pivotal role in setting the stage for confidentiality protections. Clearly articulating the measures in place to safeguard participants’ information should be a central component of informed consent discussions. Participants should be made aware of how their data will be handled, who will have access to it, and the specific steps taken to protect their confidentiality (Mootz et al., 2019). For studies involving sensitive topics, offering participants the option of anonymity or pseudonymity adds an additional layer of protection. Anonymity ensures that even the researchers cannot identify individual participants, while pseudonymity involves using a substitute name to shield identities. This provides participants with greater control over their personal information.

The storage and transmission of research data demand meticulous attention. Researchers must employ secure and encrypted systems to store and transmit data, preventing unauthorized access such as clear protocols for data access within the research team should be established, and any data shared externally must be de-identified to safeguard participants’ identities. A critical aspect of confidentiality is adhering to the principle of limited access. Only team members who absolutely need access to identifiable information should have it, minimizing the risk of unintentional breaches. Researchers should be conscientious about sharing data within the team and ensure that all members uphold strict confidentiality standards (Mootz et al., 2019).

Institutional Review Boards play a crucial role in evaluating and approving research protocols, including measures to protect participant confidentiality. Researchers should collaborate closely with IRBs to ensure that the proposed safeguards align with ethical guidelines and legal standards. IRB oversight adds an additional layer of accountability to the research process. In disseminating research findings, maintaining confidentiality extends to how results are reported. Aggregated data should be presented in a manner that prevents the identification of individual participants. Researchers must carefully balance the need for transparency with the imperative to protect the privacy of those who contributed their experiences (Mootz et al., 2019). Addressing the need for robust measures to protect participants’ confidentiality and privacy is not only an ethical responsibility but a fundamental aspect of conducting research with integrity. By prioritizing trust, tailoring informed consent, and implementing secure data practices, researchers can create an environment that respects and safeguards the privacy of participants, fostering a culture of ethical research within sensitive domains.

### 5.2 Minimizing harm: strategies to mitigate potential harm to participants during and after the research process

Ethical research in sensitive domains, such as studies involving trauma or GBV, demands a vigilant commitment to mitigating potential harm to participants. Recognizing the vulnerabilities inherent in such research, implementing comprehensive strategies is essential for ensuring participant well-being during and after the research process. The cornerstone of mitigating harm lies in a comprehensive and transparent informed consent process by clearly articulating the nature, purpose, and potential risks of the research. It is crucial to empower participants with the knowledge to make informed decisions about their involvement, and provide them with the opportunity to ask questions and clarify any concerns. Adopting trauma-informed approaches all throughout the research process, and not limiting it to the informed consent process is paramount. Sensitivity to triggers, acknowledgment of potential re-traumatization, and creating a safe and supportive environment for disclosure are critical components. Researchers must continuously reflect on the potential impact of their inquiries and interactions on participants (Sweeney et al., 2018).

In research interviews, identifying and reacting to nonverbal and verbal clues requires interpreting shifts in participants’ facial expressions, body language, and tone of voice. Researchers need to be aware of any indications that a subject is feeling triggered or uncomfortable, such as crossed arms, avoiding eye contact, altered speaking patterns, or abrupt silence (Morse, 2015). Using grounding techniques, making sure the subject feels in control, and establishing a secure, compassionate interview setting are all necessary to address these indicators. As the individual answers, researchers should modify their questions accordingly, remaining flexible and sensitive to ethical considerations all during the interview process. Interviewing effectively and respectfully requires training in active listening, empathy, and attention to participants’ boundaries.

Ensuring the confidentiality of participants is foundational for minimizing harm. It is important to Implement strong safeguards such as secure data storage, limited access, and pseudonymity or anonymity where applicable and clearly communicate these measures during the informed consent process to reassure participants of the protective steps in place. Moreover, the researcher must provide participants with information about available support resources and referrals which may include mental health services, counseling, or community organizations that specialize in trauma support. To further minimize any unintended harm, flexibility and responsive must be employed. This is done through ensuring that participants are aware of avenues for seeking assistance during or after their engagement in the research, conducting ongoing monitoring of participant well-being throughout the research process, implementing mechanisms for participants to express concerns or discomfort, and being prepared to adapt research protocols accordingly.

Following data collection, the researcher must offer debriefing sessions to participants. This provides an opportunity for them to discuss their experiences, ask questions, and address any lingering concerns. Debriefing helps participants process their involvement in the research and reinforces the supportive nature of the researcher-participant relationship. When reporting and disseminating findings, prioritizing ethical considerations to prevent potential harm is crucial. Present data in a manner that avoids sensationalism, provides a nuanced understanding of participants’ experiences, and respects the diversity of their narratives. Lastly, the researcher must ensure that the research outputs contribute positively to the broader discourse on the studied topic (Mootz et al., 2019).

Establishing mechanisms for long-term engagement and feedback is crucial for maintaining connections with participants beyond the research phase, providing ongoing support and addressing any emerging concerns. This approach ensures participants feel valued and informed about the research outcomes and its potential impact. Investing in thorough training for researchers involved in sensitive studies is essential, equipping them with the skills to recognize signs of distress, respond empathetically, and navigate challenging situations effectively. Such training should also highlight the importance of cultural relevance and a deep understanding of the unique considerations related to trauma and gender-based violence. Mitigating potential harm to participants requires a holistic and proactive approach throughout the research process and beyond. It necessitates a continuous prioritization of participant well-being, alongside a commitment from researchers to reflect on and adapt their strategies to meet evolving ethical considerations and the unique needs of those who contribute their experiences to advance knowledge in sensitive domains (Kadam, 2017).

Trauma-informed interviewing not only safeguards participants but also protects researchers by mitigating the emotional impact of these narratives. Such techniques involve understanding trauma’s effects, managing emotional responses, and maintaining professional boundaries, thus enabling researchers to handle sensitive information without harm to themselves or the interviewee (Pearlman and Mac Ian, 1995). Trauma-informed interviewing reduces the risk of vicarious trauma by equipping researchers with the knowledge and strategies to manage their emotional responses, maintain professional boundaries, and understand the effects of trauma, thereby protecting both the participants and themselves from the harmful impact of sensitive narratives.

Implementing these approaches offers a protective framework against vicarious trauma, equipping researchers with the skills needed for effective and empathetic engagement. Training in trauma-informed care is vital, emphasizing principles like safety and empowerment, to prevent re-traumatization of participants and secondary traumatic stress in researchers. Access to mental health support and continuous training in trauma awareness are essential for maintaining the well-being of researchers and the integrity of the research.

## 6 Survivor-centered methodologies in amplifying narratives of displaced individuals

In the realm of research involving displaced communities, particularly those affected by trauma, survivor-centered methodologies emerge as transformative tools that empower individuals to shape and amplify their own narratives. Survivor-centered methodologies fundamentally alter the traditional power dynamic between researchers and participants. Instead of being passive subjects, survivors become active contributors and co-creators of knowledge. This shift acknowledges their expertise on their own experiences, promoting a more equitable and respectful engagement.

Central to survivor-centered approaches is the emphasis on agency and autonomy. Displaced individuals, often marginalized and disempowered, regain control over their narratives. By actively participating in the research process, they can shape how their stories are told, ensuring that the nuances of their experiences are captured with authenticity (Thomas et al., 2022). Survivor-centered methodologies recognize and validate the diversity of displaced communities, providing a space for various voices to be heard.

Survivor-centered methodologies embrace a collaborative approach to storytelling. Instead of researchers imposing predefined narratives, participants actively contribute to shaping the research questions, methodologies, and the interpretation of findings. This collaborative process ensures that the research is culturally relevant and resonates with the lived experiences of the community. Researchers engage with participants in a manner that acknowledges the potential impact of trauma and respects individual boundaries. This sensitivity minimizes the risk of re-traumatization and fosters an environment of trust and safety.

Power imbalances within displaced communities can be further exacerbated by external research initiatives. Survivor-centered methodologies actively address these imbalances by involving local community leaders, interpreters, and other community members in the research process. This not only enhances cultural competence but also ensures that power is shared more equitably (Thomas et al., 2022). Traditional research often focuses on vulnerabilities and challenges within displaced communities. Survivor-centered methodologies, while acknowledging these challenges, also spotlight the resilience, strengths, and coping mechanisms that individuals and communities employ. This asset-based perspective contributes to a more holistic understanding of their experiences.

The ethical foundation of survivor-centered methodologies is evident in the careful consideration of informed consent processes. These methodologies prioritize transparency, ensuring that participants are well-informed about the research purpose, potential risks, and their right to control the extent of their participation (Thomas et al., 2022). This ethical approach builds trust and enhances the overall quality of the research. Survivor-centered methodologies serve as potent instruments for amplifying the narratives of displaced individuals. By reshaping power dynamics, fostering collaboration, and embracing trauma-informed principles, these approaches not only empower survivors to tell their stories but also contribute to a more nuanced and authentic understanding of the challenges and strengths within displaced communities.

### 6.1 Participatory action research: an exploration of the potential benefits and challenges of employing PAR in trauma-informed research

Participatory action research (PAR) is inherently empowering, placing survivors at the forefront of the research process empowering individuals to share their experiences on their terms, fostering a sense of agency and control over their narratives. PAR emphasizes local knowledge and context. Survivors, as active participants, contribute valuable insights into the cultural, social, and contextual dimensions of trauma. This leads to a more nuanced understanding of the experiences and needs of the community (Baum et al., 2006). The collaborative nature of PAR facilitates the co-creation of interventions that are tailor-made to the unique needs of the community. This ensures that trauma-informed interventions are not only evidence-based but also culturally relevant and aligned with the community’s values and strengths. By involving survivors in the research process, PAR promotes a sense of ownership within the community. This increases the likelihood that interventions and solutions arising from the research will be embraced, sustained, and further developed by the community itself (Baum et al., 2006). PAR is inherently ethical, as it emphasizes collaboration, transparency, and respect for participants. Survivors become active partners in decision-making processes, contributing to ethical considerations, and ensuring that the research aligns with the community’s ethical standards.

Despite the participatory ethos, power imbalances can persist. Certain individuals or groups within the community may have more influence, potentially marginalizing others. Careful attention to mitigating power differentials and ensuring equal participation is crucial. Engaging in trauma-informed research, especially when survivors actively contribute to data collection or storytelling, can be emotionally taxing. The process may evoke distressing memories, emphasizing the need for robust support mechanisms and ethical considerations to minimize potential harm (Lonbay et al., 2021). PAR is often time-intensive due to the collaborative decision-making processes. Balancing the participatory approach with the need for efficiency in research timelines can be challenging, especially when addressing urgent community needs. The effectiveness of PAR depends on the skills and resources available within the community.

In some instances, communities may lack the necessary resources or expertise, requiring external support and collaboration. Collaborative decision-making can lead to differing opinions within the community. Managing and resolving conflicts while ensuring that diverse voices are heard is a delicate balancing act in participatory research. PAR in trauma-informed research offers immense potential for empowerment, contextually rich insights, and sustainable solutions (Lonbay et al., 2021). However, it demands careful consideration of power dynamics, emotional well-being, and resource availability to navigate challenges effectively and ethically. Striking this balance ensures that the research process is not only impactful but also respectful and inclusive of the diverse voices within the community.

### 6.2 Linguistic considerations: addressing language barriers and ensuring accurate translation and interpretation

In the pursuit of understanding the experiences of gender-based violence (GBV) survivors within refugee communities, linguistic considerations emerge as a pivotal aspect of ethical research. Effective communication transcends language barriers, and thoughtful attention to linguistic nuances is essential for conducting trauma-informed interviews that respect the diverse cultural backgrounds and experiences of GBV survivors. GBV survivors from refugee communities may carry with them diverse linguistic expressions and cultural nuances that shape their understanding and articulation of traumatic experiences. A culturally sensitive approach to language recognizes the richness of expression inherent in different languages and dialects. The use of trauma-informed language, which is sensitive to the potential triggers and emotional impact of certain terms, is crucial. This involves careful consideration of the words and phrases used during interviews to create a safe and supportive environment for survivors to share their experiences (Sweeney et al., 2018).

In cases where language proficiency is a barrier, interpreters play a critical role as cultural brokers. The selection of interpreters who are not only fluent in the language but also culturally competent is essential. They serve as conduits for accurate communication, ensuring that the survivor’s narrative is faithfully conveyed. Idiomatic expressions and cultural signifiers carry profound meanings within a community. Researchers must be attuned to these linguistic nuances, recognizing that certain words or phrases may convey deeper cultural or contextual meanings that impact the survivor’s narrative. A nuanced understanding ensures that interpretations are not oversimplified. The interview process should not inadvertently re-traumatize survivors. Certain language choices or questions may unintentionally evoke distressing memories. Linguistic considerations involve crafting questions and responses that are sensitive to potential triggers, minimizing the risk of re-traumatization during the interview (Squires, 2009).

Moreover, refugee communities are often linguistically diverse. Thus, understanding the multilingual dynamics within the community is crucial for ensuring inclusive research. Researchers should be aware of the various languages spoken and be prepared to accommodate different linguistic needs, acknowledging that language proficiency varies among survivors.

Language is a powerful tool for building trust. Using the survivor’s preferred language and demonstrating linguistic competence fosters a sense of understanding and trust. This is particularly important in establishing rapport, as survivors may be more inclined to share their experiences when they feel their language and culture are respected. If the research involves translation of materials or findings, maintaining the accuracy and fidelity of translations is crucial. Ethical considerations extend to the translation process, ensuring that the essence of survivors’ narratives is preserved without distortion. The importance of linguistic considerations in interviewing GBV survivors from refugee communities cannot be overstated. A thoughtful and culturally informed approach to language is not merely a technical aspect of research; it is a fundamental dimension of ethical research practices that honors the diversity, experiences, and cultural richness of GBV survivors within refugee contexts.

## 7 Recommended ways forward

The researcher’s role extends beyond the technicalities of data collection and analysis. It is a multifaceted responsibility that demands a commitment to ethical, trauma-informed, and culturally sensitive methodologies. By actively embodying these principles, researchers not only enhance the validity of their findings but also contribute to the well-being and empowerment of survivors from displaced communities, fostering a research environment rooted in respect, empathy, and ethical integrity.

### 7.1 The need for researchers to undergo training in trauma-informed approaches

In the realm of research, particularly in areas that delve into sensitive topics such as trauma, GBV, or refugee experiences, the need for researchers to undergo training in trauma-informed approaches is paramount. This imperative arises from the recognition that research endeavors have the potential to influence the lives and well-being of individuals, and employing trauma-informed methodologies is crucial for ethical, respectful, and impactful investigations. Trauma is a multifaceted experience with diverse manifestations. Researchers need a nuanced understanding of how trauma may intersect with various aspects of individuals’ lives, such as culture, identity, and socioeconomic factors. Training equips researchers with the necessary sensitivity to recognize and respond to the complexity of trauma experiences. Research interactions have the potential to evoke distressing memories or emotions for participants, especially when discussing topics like GBV or displacement. Training in trauma-informed approaches enables researchers to navigate these discussions with care, minimizing the risk of re-traumatization and prioritizing the well-being of participants.

Trauma survivors may be hesitant to disclose their experiences due to fear, shame, or mistrust. Training equips researchers with the skills to create safe and supportive environments that encourage open communication. This involves understanding the power dynamics inherent in research interactions and fostering trust through empathetic and non-judgmental approaches. Researchers need to be attuned to potential triggers that may emerge during interviews or interactions. Training enables them to recognize signs of distress and respond appropriately, ensuring that the research process does not inadvertently exacerbate participants’ emotional vulnerabilities. Common signs include emotional distress such as anxiety, fearfulness, and depression, physical symptoms like headaches and fatigue, avoidance behaviors, flashbacks, hyper arousal, social withdrawal, substance use, and self-harming behaviors. Researchers should be trained to recognize these signs, establish support protocols, and ensure a safe and confidential environment to conduct ethical research with GBV survivors. Cultural competence is integral to trauma-informed research, especially when working with diverse populations. Training provides researchers with the skills to navigate cultural nuances, respect diverse perspectives, and adapt methodologies to align with the cultural backgrounds of participants.

Trauma-informed approaches guide researchers in crafting ethical and non-intrusive questions. Here are some examples of how researchers can craft ethical and non-intrusive questions: Instead of asking directly about traumatic events, researchers might inquire about participants’ general experiences and well-being. For example: “Can you tell me about your experiences with relationships and support networks?” or “How have past experiences influenced your feelings about trust and safety?”. Researchers can use open-ended questions that allow participants to share their experiences in their own words without feeling pressured to disclose specific details:

”Can you describe a time when you felt supported or understood during a difficult situation?” or “What strategies have helped you cope with challenges in your life?” Avoiding leading or triggering language is crucial. Instead of assuming specific details about the participant’s experience, researchers can use neutral language: “Can you tell me about any challenges you’ve faced in your relationships or personal life?” or “How do you typically cope with stress or difficult emotions?” Providing options for participants to decline answering sensitive questions or to take breaks during the interview can empower them to maintain control over their participation: “If there’s anything you’re not comfortable discussing, please let me know, and we can move on.” or “Feel free to take a break or pause the interview at any time if you need to.” Incorporating validating and supportive statements throughout the interview process can help participants feel heard and respected: “Thank you for sharing your experiences with me. It’s important for me to understand your perspective.” or “I appreciate your willingness to participate in this research. Your insights are valuable.” The following table sheds further light on questions to avoid and how the researcher can reframe them.

**Table:** 

Questions to avoid	Trauma Informed Reframing	Rationale/Benefits
“Why did you …?” or “Why didn’t you …?”	“When (a specific event happened), what were your feelings and thoughts?” or “Are you able to tell more about what happened when…?”	asking “why” questions will be challenging for survivors to answer. Furthermore, it will increase their guilt feelings, and, most importantly, it can be perceived by survivors as blaming them for not acting in a certain way. Therefore, starting the question with “Are you able to tell me what happened when …” instead of “Why you did or did not …” will remove the pressure on survivors to explain why they did or did not act in a certain way.
How did it happen?	Always ask about what happened and not how it happened.	Asking about how a certain SGBV incident happened, will put a lot of pressure on survivors, and they may think that the interviewer wants to know details about the incident. Secondly, details can be intrusive and unnecessary for the purposes of the interview. Asking about what happened can be more relevant, and can elicit more other relevant information
Questions that ask about chronological order of events. Ex: What happened First? What did you do next?	Examples: “Where would you like to start?” or “Would you tell me what you are able to remember about your experience? You can start where it feels natural.”	Reframing the questions with the free recall technique and opening with “What are you able to…” can reduce the pressure on the survivor to recall specifics given the impact of trauma on memory. This technique can also help the interviewer in asking follow up questions that can further clarify or expand the account.

Training helps researchers strike a balance between gathering comprehensive data and respecting participants’ boundaries, ensuring that the interview process is both thorough and ethically sound. Confidentiality is a cornerstone of trauma-informed research. Training emphasizes the importance of robust measures to protect participants’ confidentiality and privacy, instilling in researchers the ethical imperative to safeguard the information shared by participants. Engaging with traumatic narratives can impact researchers’ well-being. Training not only equips researchers with the skills to support participants but also emphasizes the importance of self-care and resilience-building strategies to mitigate the potential emotional toll on researchers themselves. The need for researchers to undergo training in trauma-informed approaches is rooted in the ethical responsibility to conduct research with sensitivity, empathy, and respect for the well-being of participants. By investing in such training, researchers contribute to the advancement of knowledge while upholding the ethical principles that underpin meaningful and impactful research in areas that intersect with trauma.

### 7.2 The advantages of collaborating with local organizations and community leaders to ensure cultural relevance and sensitivity

In the landscape of research on GBV among refugee communities, the advantages of collaborative partnerships with local organizations and community leaders are paramount. Such collaborations serve as a cornerstone for ensuring cultural relevance, sensitivity, and the ethical conduct of research, offering multifaceted benefits that contribute to the authenticity and impact of the study. Local organizations and community leaders bring inherent cultural competence and contextual insight. Their intimate understanding of the community’s norms, values, and nuances provides researchers with invaluable guidance in crafting research approaches that are culturally relevant and respectful of local sensitivities.

Trust is foundational in research, particularly when investigating sensitive topics like GBV. Collaborating with local organizations and community leaders facilitates the establishment of trust and rapport with the refugee community. These trusted intermediaries can vouch for the credibility of the research, easing participants’ apprehensions and encouraging open and honest dialogue. Language can be a significant barrier in research. Local organizations and community leaders, often bilingual or multilingual, can bridge linguistic gaps, ensuring effective communication between researchers and participants. This enhances the quality and accuracy of data collection, preventing potential misinterpretations that may arise from language differences. Collaborators from the local community offer insights that go beyond language proficiency. They can contribute to the design of research instruments, ensuring that questions are culturally sensitive, respectful, and relevant. This collaborative approach leads to a more accurate representation of GBV experiences within the cultural context of the refugee community.

Collaborative partnerships enable the adoption of community-centered research methodologies. Local organizations and community leaders can guide the implementation of approaches that prioritize the well-being and preferences of the community, ensuring that research processes align with local cultural norms and practices. Local collaborators facilitate access to the community and enhance participant engagement. Their established networks and relationships within the community streamline the recruitment process, making it more inclusive and allowing researchers to reach a broader and more representative sample of GBV survivors among refugees. Ethical considerations, particularly around informed consent, gain depth through local collaboration. Community leaders can assist in developing culturally appropriate consent processes, ensuring that participants fully comprehend the research goals, potential risks, and their rights, thus upholding the ethical standards of the study.

Collaborative research has the potential to leave a lasting impact on the community. By involving local organizations and leaders, research efforts can contribute to community empowerment, skill-building, and awareness. This not only enriches the community but also fosters sustainability beyond the duration of the research project. The advantages of collaborating with local organizations and community leaders in research on GBV survivor refugees extend far beyond logistical facilitation. These collaborations foster a holistic understanding of the community’s dynamics, enhance the ethical conduct of research, and contribute to the creation of knowledge that is culturally relevant, sensitive, and beneficial to the refugee community. The reciprocity inherent in such partnerships underscores the importance of community collaboration as an ethical and effective approach in research endeavors.

## 8 Conclusion

In the realm of qualitative research with survivors from displaced communities, the role of researchers is pivotal not only in gathering data but, more importantly, in fostering ethical and trauma-informed methodologies. Recognizing the unique vulnerabilities of displaced populations, researchers bear the responsibility of shaping research practices that prioritize the well-being, dignity, and agency of survivors. This proactive stance is essential for cultivating a research environment that is not only methodologically robust but also ethically sound and trauma-informed.

Researchers must first cultivate a profound understanding of the impact of trauma. This entails delving into the psychological, emotional, and social consequences of displacement and violence. Such awareness is foundational for crafting methodologies that respect the sensitivities and boundaries of survivors, minimizing the risk of re-traumatization. Researchers are the architects of the research design. They play a crucial role in embedding trauma-informed principles within the methodology. This involves creating safe spaces for disclosure, employing non-triggering language, and incorporating flexibility to adapt to participants’ comfort levels during interviews. Building trust is paramount in research with displaced communities. Researchers act as facilitators in establishing a rapport with survivors. This involves transparent communication, active listening, and cultural humility to ensure that survivors feel heard, understood, and respected throughout the research journey.

Cultural relevance is an ethical imperative. Researchers must be attuned to cultural nuances, recognizing the diversity within displaced communities. This sensitivity guides the formulation of interview questions, ensuring that they resonate with the cultural and contextual realities of survivors, thus contributing to the richness and authenticity of the data. Researchers are catalysts for collaboration with local organizations and community leaders. Such partnerships are instrumental in navigating cultural intricacies, providing linguistic support, and facilitating community engagement. Researchers must actively seek and respect the guidance of local partners, ensuring that the research is contextualized and ethically grounded.

The informed consent process is a linchpin of ethical research. Researchers play a central role in ensuring that the process is comprehensive, culturally sensitive, and trauma-informed. This involves clearly articulating the purpose of the research, potential risks, and the voluntary nature of participation, while respecting survivors’ autonomy. Rigorous research design is crucial for generating credible findings. However, researchers must strike a delicate balance between methodological rigor and the sensitivity required when working with trauma survivors. Flexibility in approaches, reflexivity, and ongoing ethical reflections are essential aspects of this balancing act. Researchers carry the responsibility of disseminating findings ethically. This involves presenting data in a manner that respects the dignity and privacy of survivors, avoiding sensationalism, and contributing nuanced insights to the broader discourse on displacement and trauma. Researchers should be advocates for ethical reporting within academic and public spheres.

An example of this approach being successfully implemented with survivors of sexual violence from migrant communities in Lebanon is illustrated in a paper published in *Frontiers in Sociology* by one of the authors of this piece (Diab et al., 2023). In this study, the largest study of sexual violence ever carried out in Lebanon, a trauma-informed (participatory) approach was adopted to qualitatively explore the experiences of nearly 1,000 women from diverse migrant communities. The trained interviewers were not only from the same communities as the women in question but were also survivors of various forms of sexual violence themselves. They spoke to each interviewee in their native language, established trust with the participants, and most importantly, overcame linguistic barriers to ensure that survivors could authentically and openly discuss their experiences.

## Data availability statement

The original contributions presented in the study are included in the article/supplementary material, further inquiries can be directed to the corresponding author.

## Author contributions

JD: Writing–review and editing, Writing–original draft, Supervision, Methodology, Formal Analysis, Conceptualization. DA-A: Writing–review and editing, Methodology, Formal Analysis, Conceptualization.
